# Preparation of Pickering emulsions through interfacial adsorption by soft cyclodextrin nanogels

**DOI:** 10.3762/bjoc.11.257

**Published:** 2015-11-30

**Authors:** Shintaro Kawano, Toshiyuki Kida, Mitsuru Akashi, Hirofumi Sato, Motohiro Shizuma, Daisuke Ono

**Affiliations:** 1 Biomaterial and Commodity Chemicals Research Division, Osaka Municipal Technical Research Institute, 1-6-50 Morinomiya, Joto-ku, Osaka 536-8553, Japan; 2 Department of Applied Chemistry, Graduate School of Engineering, Osaka University, Suita, Osaka 565-0871, Japan

**Keywords:** crosslinked cyclodextrin polymer, interfacial adsorption, nanogel, Pickering emulsion

## Abstract

**Background:** Emulsions stabilized by colloidal particles are known as Pickering emulsions. To date, soft microgel particles as well as inorganic and organic particles have been utilized as Pickering emulsifiers. Although cyclodextrin (CD) works as an attractive emulsion stabilizer through the formation of a CD–oil complex at the oil–water interface, a high concentration of CD is normally required. Our research focuses on an effective Pickering emulsifier based on a soft colloidal CD polymer (CD nanogel) with a unique surface-active property.

**Results:** CD nanogels were prepared by crosslinking heptakis(2,6-di-*O*-methyl)-β-cyclodextrin with phenyl diisocyanate and subsequent immersion of the resulting polymer in water. A dynamic light scattering study shows that primary CD nanogels with 30–50 nm diameter assemble into larger CD nanogels with 120 nm diameter by an increase in the concentration of CD nanogel from 0.01 to 0.1 wt %. The CD nanogel has a surface-active property at the air–water interface, which reduces the surface tension of water. The CD nanogel works as an effective Pickering emulsion stabilizer even at a low concentration (0.1 wt %), forming stable oil-in-water emulsions through interfacial adsorption by the CD nanogels.

**Conclusion:** Soft CD nanogel particles adsorb at the oil–water interface with an effective coverage by forming a strong interconnected network and form a stable Pickering emulsion. The adsorption property of CD nanogels on the droplet surface has great potential to become new microcapsule building blocks with porous surfaces. These microcapsules may act as stimuli-responsive nanocarriers and nanocontainers.

## Introduction

Pickering emulsions, which are emulsions stabilized by colloidal particles instead of conventional low-molecular-weight surfactants [1], are formed through the adsorption of colloidal particles at an oil–water interface to give stable emulsion droplets. Particle adsorption, which depends on the wettability of the particles against the two fluids, is related to the contact angle at the oil–water interface [2].

Although many reports have used inorganic [3–4] or organic particles [5–6] as Pickering emulsifiers, soft microgels, which are colloidal particles composed of swollen crosslinked polymers, have recently been demonstrated as Pickering emulsifiers with pH or thermo-responsive properties [7–9]. Such emulsions have potential in pharmaceutical, food, and cosmetic products. Moreover, emulsions stabilized by stimuli-responsive soft microgels should be applicable as templates to fabricate functional materials such as hollow permeable microcapsules. With regard to biomedical and pharmaceutical fields, emulsifiers derived from natural polymers such as saccharides are attractive compared to Pickering emulsifiers derived from synthetic polymers.

Cyclodextrins (CDs) are cyclic oligosaccharides, which have subnanometer-sized cavities where guest molecules with an appropriate size and shape are incorporated [10]. CDs have been reported to work as emulsion stabilizers [11–14]. Previous studies have shown that CDs can form surface-active inclusion complexes with oil molecules at the oil–water interfaces that can stabilize emulsions, although they do not alter the surface tension of water alone [15].

Only a few papers have reported Pickering emulsions stabilized by CD inclusion complexes. For example, Inoue et al. prepared oil-in-water (O/W) emulsions with α-, β- and γ-CDs using an *n*-alkane/water system [12]. They reported that the most stable emulsion is formed by β-CD–oil complexes when the contact angles are close to 90° at the oil–water interface. Davarpanah et al. examined the relationship between the stability of O/W Pickering emulsions formed through complexation of β-CD with select oil solvents and the interfacial tension at the oil–water interface [13]. Mathapa et al. described the effect of the particle size formed with the CD–oil complex on the stability of the Pickering emulsion [14]. They also reported that “CD colloidosomes” composed of the CD–oil complexes microparticles can be formed by an emulsion template and subsequent core oil removal. These previous works have been limited to preparing Pickering emulsions via the formation of CD–oil complexes at the oil–water interface. Thus, emulsion formation requires a high concentration of CDs and depends on the type of oil solvent.

Our research focuses on a new class of Pickering emulsifiers based on nanometer-sized hydrogel nanoparticles composed of crosslinked CD polymers (hereafter CD nanogels), which have a surface-active property and form stable emulsions at the oil-water interface. Reports on CD nanogels have been increasing in terms of drug delivery systems using the nanoporous cavities of the CDs and hydrogel networks, which can effectively store and release molecules [16–17]. However, to the best of our knowledge, the use of CD nanogels as Pickering emulsifiers has yet to be reported.

We have previously reported urethane-crosslinked CD polymers, which were prepared by reacting heptakis(2,6-di-*O*-methyl)-β-cyclodextrins (DM-β-CDs) with aromatic diisocyanates such as 4,4’-methylenebis(phenyl isocyanate) (MDI) and 1,4-phenylene diisocyanate (PDI) [18]. Although MDI- or PDI-crosslinked DM-β-CD polymers bearing a [MDI or PDI]/[DM-β-CD] feed ratio of more than three has a lipophilic nature, they show a poor hydrophilicity. Controlling the degree of crosslinking should provide an appropriate balance between the hydrophilicity and hydrophobicity, generating an amphiphilic crosslinked CD polymer, which can be dispersed in water as well as in nonpolar solvents. This amphiphilic polymer should realize water-swellable hydrogel nanoparticles containing CDs (CD nanogels). In this paper, we describe the preparation of Pickering emulsions using CD nanogels composed of crosslinked DM-β-CD polymers in water.

## Results and Discussion

### Preparation and characterization of the CD nanogels

**Figure 1:**
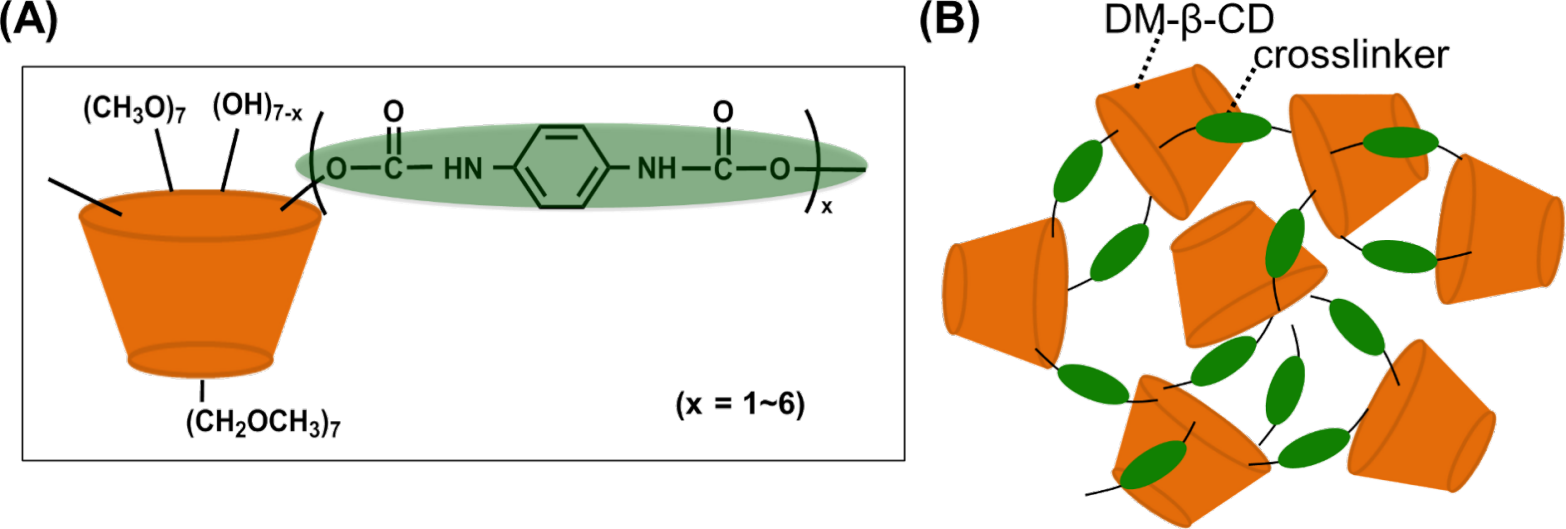
(A) Chemical structure and (B) schematic illustration of DM-β-CD/PDI polymer.

Nanometer-sized CD nanogels were prepared by crosslinking DM-β-CD with PDI and subsequent immersion of the resulting polymers in water (Figure 1). The crosslinking reaction was performed at 70 °C for 24 h (the crosslinker/DM-β-CD feed ratio = 3/1). The resulting crosslinked DM-β-CD/PDI polymer is water soluble. The polymer product was purified by dialysis (molecular weight cut off = 10,000) against water.

A powder of the DM-β-CD/PDI polymer obtained by freeze-drying was easily redispersed in water with the aid of ultrasonication. Previously, we prepared a urethane-crosslinked DM-β-CD/PDI polymer at a higher crosslinker/DM-β-CD feed ratio (>5.0). The resulting polymer forms a submicrometer-sized particle and does not disperse in water [18]. These results reveal that controlling the crosslinking degree gives a polymer with a good colloidal stability in water.

**Figure 2:**
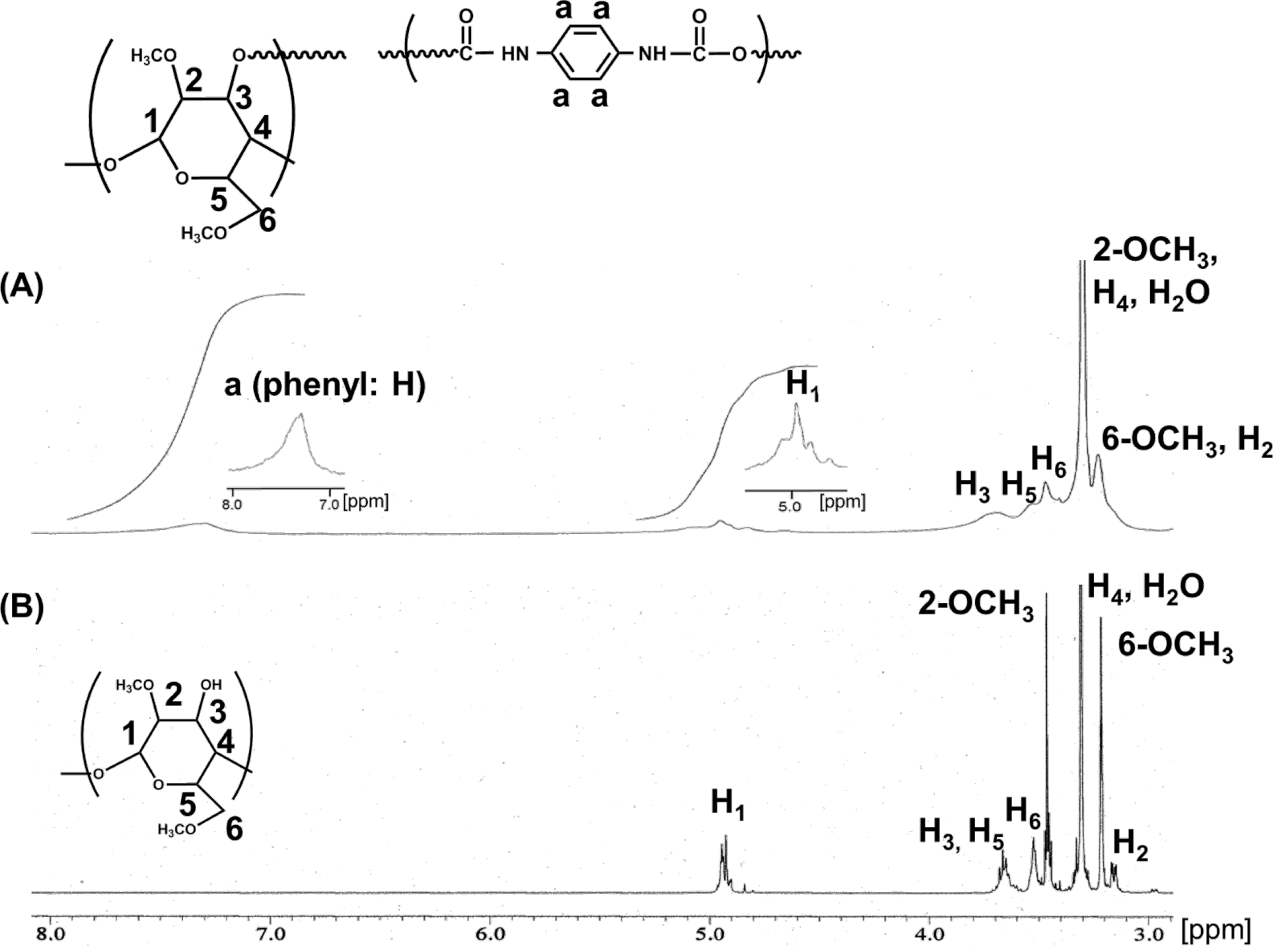
^1^H NMR spectra of (A) DM-β-CD/PDI polymer and (B) DM-β-CD in (CD_3_)_2_SO.

Figure 2 shows the ^1^H NMR spectra of the DM-β-CD/PDI polymer and DM-β-CD. The peaks of the DM-β-CD and 1,4-phenylene dicarbamyl (PDC) segments are broadened due to the restricted movement of the network linkage of the polymer. The integral ratio of the peaks for the phenyl groups in PDC to the peaks of H_1_ protons of DM-β-CD indicates that the PDC/DM-β-CD ratio is two. The appearance of C=O stretching bands from urethane (1714 cm^−1^) in the FTIR spectrum (Supporting Information File 1, Figure S1) confirms that a urethane-crosslinked polymer is formed. In addition, a O–H stretching band at 3309 cm^−1^ shows that free OH groups remain in the polymer.

The surface charge of the DM-β-CD/PDI polymer nanogels was characterized by measuring the zeta potential of the aqueous solution through electrophoretic analysis at various pH levels. The zeta potential is approximately +2.0 to +4.0 mV for pH 5.0 to 9.0, suggesting that the nanogels have almost neutral surface charges (Figure S2, Supporting Information File 1). Thus, the following study was carried out using CD nanogel solutions without pH control.

**Figure 3:**
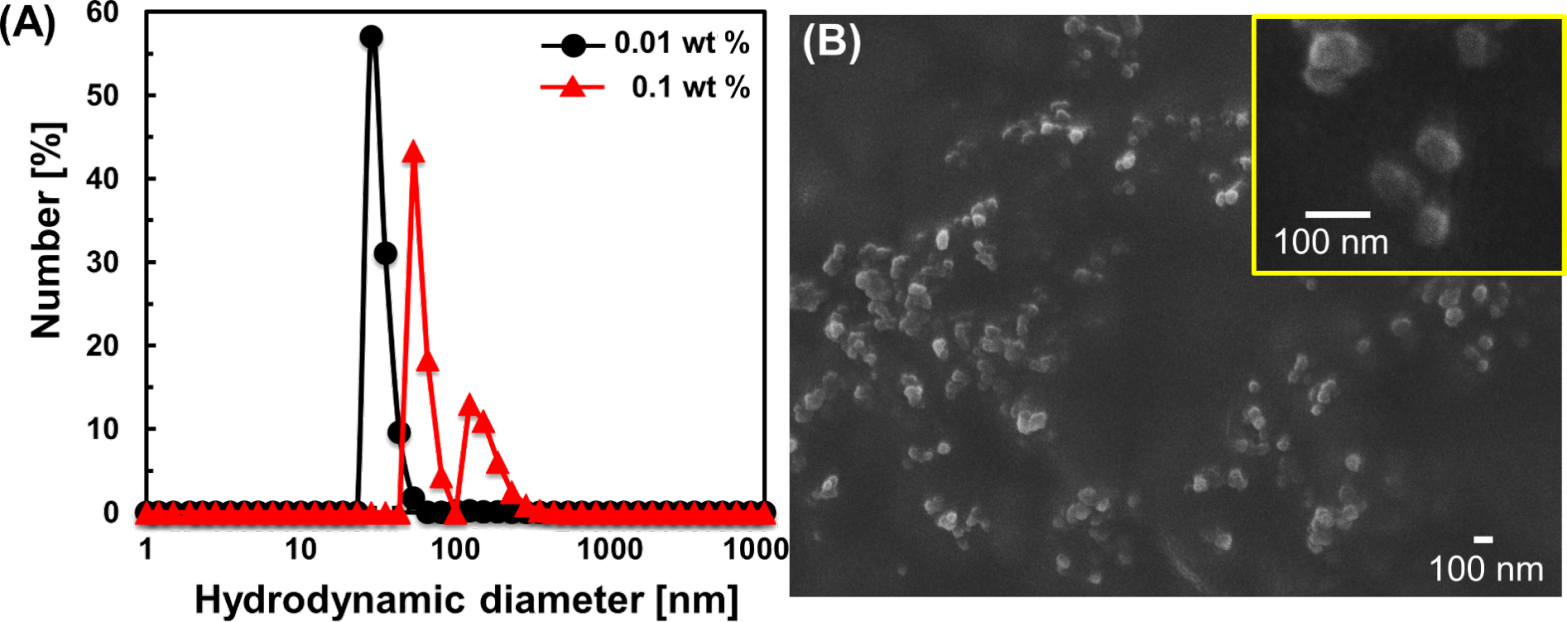
(A) Number-averaged particle size distributions of DM-β-CD/PDI nanogels measured by DLS at concentrations of 0.01 and 0.1 wt % in water. (B) SEM image of DM-β-CD/PDI nanogels. Inset shows the magnified image.

The hydrodynamic diameter and size distribution of the CD nanogels were studied using dynamic light scattering (DLS). Figure 3A shows the DLS data of CD nanogel obtained after redispersion of the freeze-dried DM-β-CD/PDI polymer powder in water. The concentration of the CD nanogel was adjusted to 0.01 and 0.1 wt %. At a 0.01 wt % concentration, a unimodal size distribution occurs with a peak at 30 nm diameter. When the concentration of the CD nanogel is increased to 0.1 wt %, a bimodal size distribution is observed and the major peak shifts from a 30 nm (at 0.01 wt %) to 50 nm (at 0.1 wt %) diameter, and a new peak appears at 120 nm diameter. An increase in the particle size of the primary CD nanogel and the appearance of a new peak at a larger diameter (120 nm) may be due to the self-assembly of the primary CD nanogels at 0.1 wt % concentration in water.

The scanning electron microscopy (SEM) measurement was carried out in order to observe the self-assembled nanogel structures. Prior to the observation, the CD nanogel was immediately frozen, using liquid nitrogen, and freeze-dried. The diameters of the spherical particles range from 50 to 100 nm (Figure 3B), confirming the formation of self-assembled CD nanogels in water. The transmission electron microscopy (TEM) images demonstrate that the self-assembled CD nanogels consist of primary CD nanogel cores (Figure S3, Supporting Information File 1).

To investigate the surface activity of CD nanogels, the surface tension of the aqueous solution was measured by the pendant drop method. The surface tensions of the aqueous solutions of DM-β-CD and the DM-β-CD/PDI nanogels at different concentrations are shown in Table 1 and Figure S4 (Supporting Information File 1). The surface tension of the DM-β-CD/PDI nanogel solution remarkably decreases when the concentration increases in the range from 1.0 × 10^−3^ to 4.0 × 10^−2^ wt %, indicating that the DM-β-CD/PDI nanogels have the ability to lower surface tension through the adsorption at the air–water interface. Then, the surface tension reaches a plateau, showing that the nanogels self-aggregate in water. A critical aggregation concentration (CAC) was estimated to be 4.0 × 10^−2^ wt % from the breakpoint of the surface tension vs concentration (on log-scale) curve (Figure S4, Supporting Information File 1). Comparison of the surface tension between the DM-β-CD/PDI nanogel solution and the DM-β-CD solution reveals that DM-β-CD/PDI nanogels show a greater ability to lower surface tension as compared to that of DM-β-CD in the concentration range examined. Moreover, an aqueous solution containing CD nanogels gradually becomes opaque as the CD nanogel concentration increases (>0.1 wt %) possibly due to self-aggregation.

**Table 1:** Surface tensions of aqueous solutions of DM-β-CD/PDI nanogels and DM-β-CD at different concentrations.

	DM-β-CD/PDI polymer		DM-β-CD	

Concentration [wt %]	Surface tension^a^ [mN/m]	S.D.^b^	Surface tension^a^ [mN/m]	S.D.^b^

1.0 × 10^−3^	62.9	2.3	64.4	0.90
1.0 × 10^−2^	56.8	0.5	62.4	1.6
5.0 × 10^−2^	54.5	0.8	58.5	0.9
1.0 × 10^−1^	54.4	1.2	57.5	0.8
5.0 × 10^−1^	54.2	1.1	56.1	0.6

^a^Measured after allowing to stand for 1 h. ^b^Standard deviation (S.D.) was calculated from the average of the runs.

### Formation of Pickering emulsions

**Figure 4:**
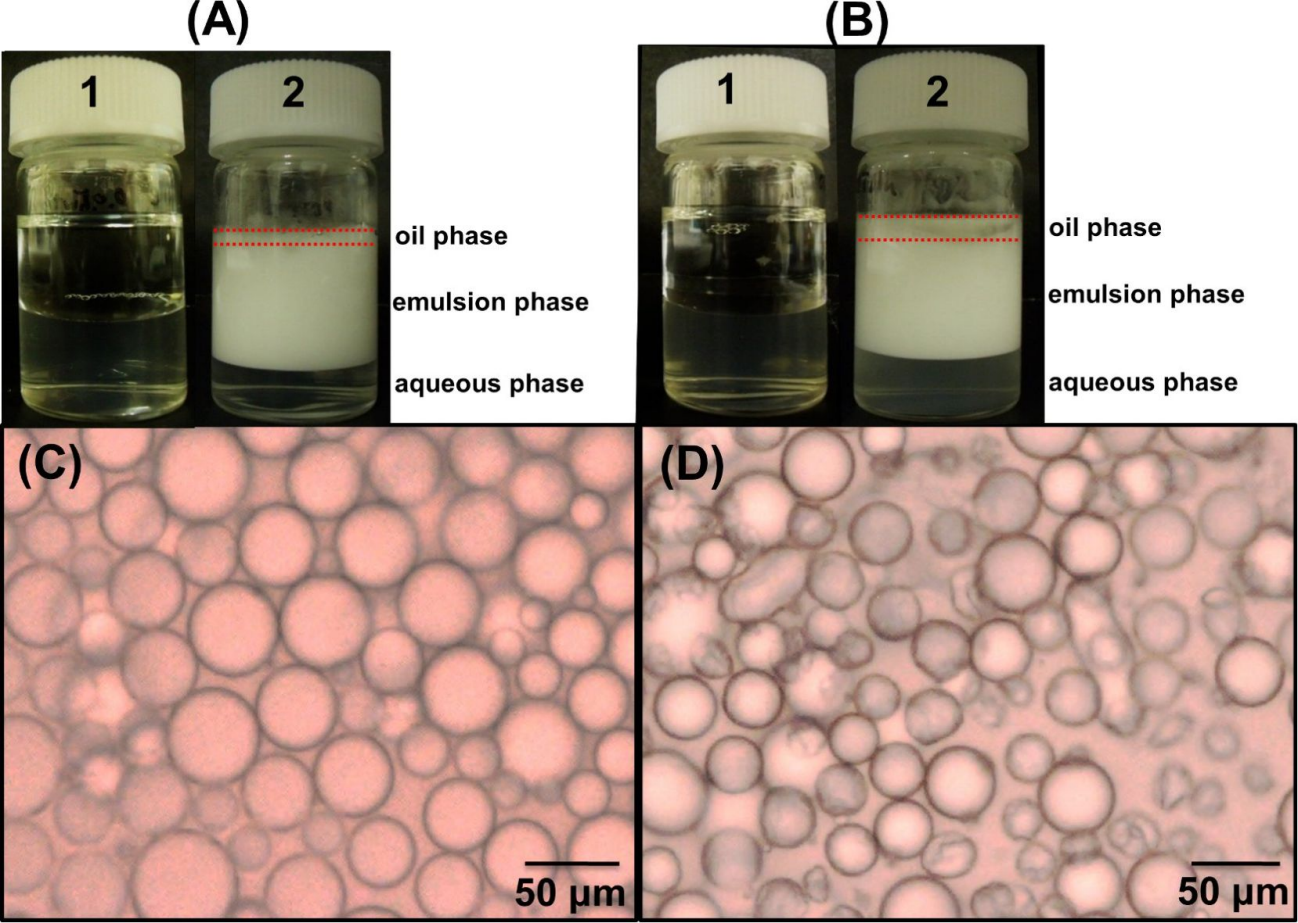
(A,B) Digital photograhs of (1) the initial DM-β-CD/PDI nanogel (0.1 wt %) with (A) *n*-dodecane or (B) toluene added directly to the vial and (2) the *n*-dodecane- or toluene-in-water emulsion stabilized by the DM-β-CD/PDI nanogel after standing for 24 h. Red dotted lines show oil phase boundaries. (C,D) The corresponding optical microscope image for the (C) *n*-dodecane- or (D) toluene-in-water droplets stabilized with the DM-β-CD/PDI nanogel.

The DM-β-CD/PDI nanogel functions as a good Pickering emulsifier when a mixture of the aqueous solution (0.1 wt %) with either *n*-dodecane (Figure 4A) or toluene (Figure 4B) is homogenized at 8000 rpm for 1 min. A milky emulsion phase is produced after standing for 24 h when a 50:50 oil/aqueous volume ratio (denote Φ_oil_ = 0.5) is employed.

In each case, stable creaming is visually observed without further phase separation where the lower phase is composed of the CD nanogel aqueous solution. The drop test confirmed the presence of an oil-in-water (denote O/W) emulsion. Upon adding a drop of the emulsion to water, the emulsion disperses well in water. The volume fraction of the emulsion phase was investigated by monitoring the height of the upper oil, emulsion, and lower aqueous phases. When *n*-dodecane was used as an oil phase, the volume fractions for the lower aqueous, emulsion, and the upper oil phases are 0.25, 0.69, and 0.06, respectively (Figure 4A-2). In the case of toluene, the volume fractions for the lower aqueous, emulsion, and the upper oil phases are 0.29, 0.62, and 0.09, respectively (Figure 4B-2). These results show a slight phase separation of the oil from the emulsion phase. It should be noted that reducing a volume ratio of oil (Φ_oil_ = 0.3) does not result in a phase separation for either oil (data not shown).

Optical microscopy observation confirmed the presence of dispersed oil droplets for both O/W emulsions as shown in Figure 4C (for *n*-dodecane) and 4D (for toluene). The emulsion droplets are slightly larger for the *n*-dodecane (35 ± 10 μm) compared to those of toluene (24 ± 5.1 μm). The mean droplet sizes and the corresponding emulsion volume fraction should be related to the Hansen solubility parameter of the oil [19]. Organic solvents possess a solubility parameter, which is manifested from dispersion forces (δ_d_), polarity (δ_p_), and hydrogen bonding forces (δ_h_). Toluene has a relatively higher contribution in terms of δ_p_ and δ_h_ compared to *n*-dodecane; these parameters may result in a higher affinity for the interface of toluene and water compared to that of *n*-dodecane and water. Thus, the droplet sizes are slightly smallerer for toluene than that of *n*-dodecane [13], while the emulsion volume fraction, which is correlated to the emulsion stability, is higher for *n*-dodecane than that of toluene. Previous results on the effect of the oil type when preparing a Pickering emulsion have shown that a relatively nonpolar oil favors the formation of an O/W type emulsion, while a polar oil favors the formation of a W/O type emulsion [20].

**Figure 5:**
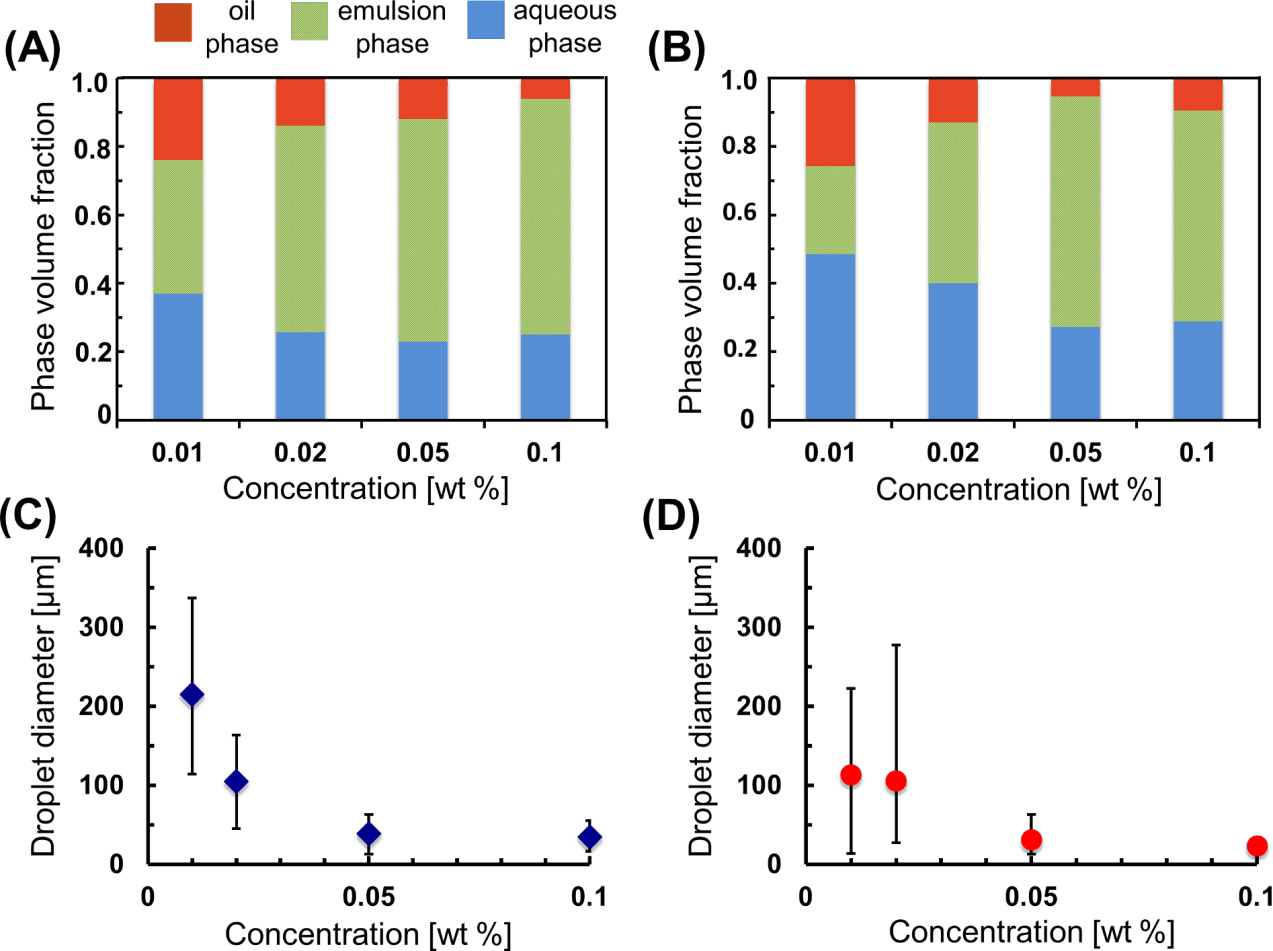
(A,B) Phase volume fractions and (C,D) droplet diameters of *n*-dodecane-(A) or toluene- (B) in-water emulsion stabilized by the DM-β-CD/PDI nanogel at various nanogel concentrations.

The stabilities of the *n*-dodecane- and toluene-in-water emulsions formed by the DM-β-CD/PDI nanogel were studied by varying the CD nanogel concentration (Figure 5A,B). The relationship between the oil droplet diameter and the corresponding CD nanogel concentrations (Figure 5C,D) was also examined. In both oils, when the CD nanogel concentration increases from 0.01 to 0.1 wt %, the emulsion phase volume fractions increase, indicating that the Pickering emulsion becomes more stable. In a concentration range from 0.05 to 0.1 wt %, the change in the emulsion volume fractions is negligible for both oils, suggesting that 0.05 wt % concentration of the CD nanogel is sufficient to inhibit coalescence of the emulsions. Therefore, the mean droplet diameters remain constant in the concentration range from 0.05 to 0.1 wt %. In the case of the 0.01 wt % concentration, the initial emulsion volume fractions are lower and their droplet diameters are higher, indicating a remarkable decrease in the emulsion stabilities for both oils. However, the creamed emulsion phases never become clear, even at a CD nanogel concentration as low as 0.01 wt %.

It should be noted that the DM-β-CD/PDI nanogel realizes the formation of a Pickering emulsion at ten times lower concentrations compared to previous emulsifiers using CDs [12–14]. Moreover, conventional methods depend on the formation of inclusion complexes between the CDs and oil molecules at an interface. The findings herein demonstrate that CD nanogels have efficient adsorption properties at oil–water interfaces to stabilize emulsions. The adsorption properties of CD nanogels, which consist of hydrophilic DM-β-CD and lipophilic benzene parts, may be affected by their hydrophilic and lipophilic balance of the nanogels.

### Identification of the CD nanogel assembly at the interface

**Figure 6:**
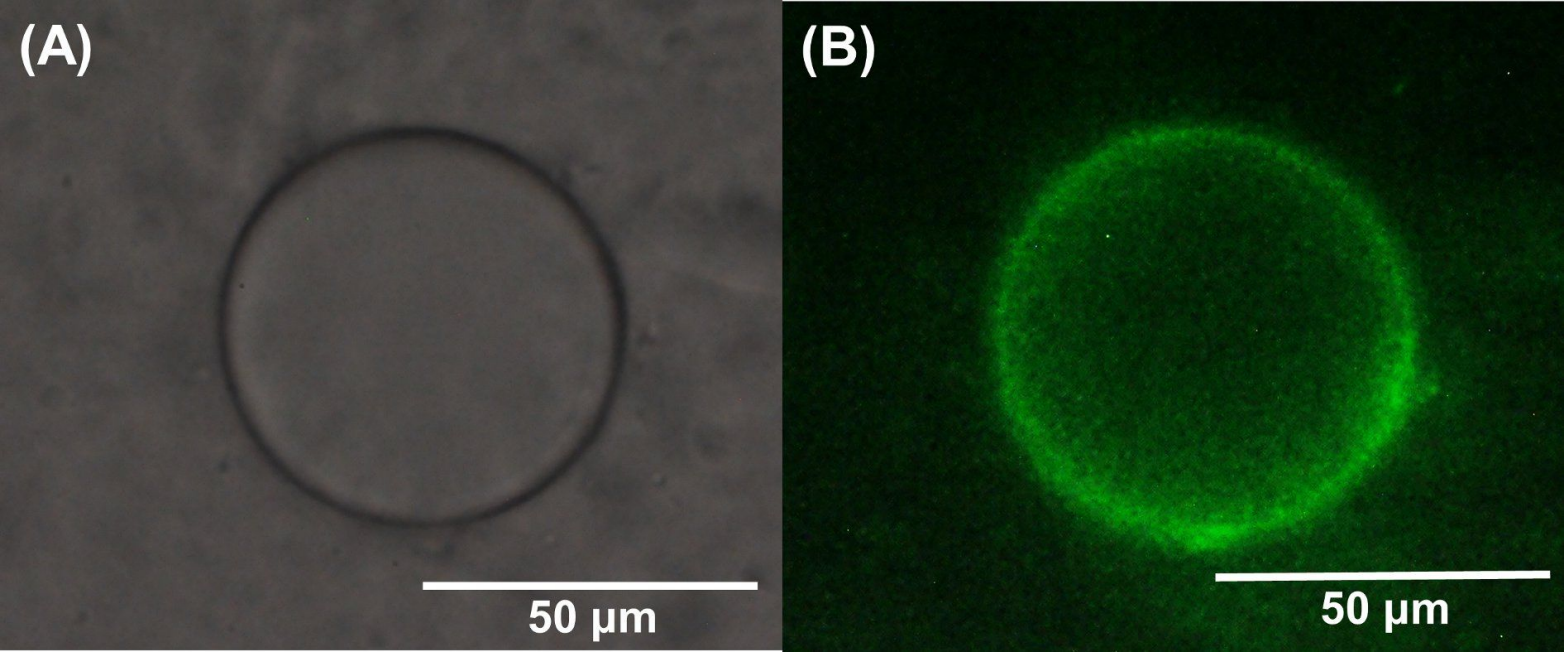
(A) Optical and (B) fluorescence micrographs of the *n*-dodecane-in-water emulsion stabilized by the FITC-labeled DM-β-CD/PDI nanogel before (A) and after (B) observations with the filter sets (excitation wavelength: 470 nm and emission wavelength: >510 nm).

Adsorption of a CD nanogel at the oil–water interface in the emulsion phase was identified using a fluorescent dye-labeling method. The CD nanogel was labeled with fluorescein isothiocyanate (FITC) in the aqueous phase before emulsification. A Pickering emulsion was prepared by mixing the aqueous dispersion containing FITC labeled-CD nanogels (0.1 wt %) with *n*-dodecane (Φ_oil_ = 0.1) and subsequent shaking for 1 min. After dilution with water, the emulsion was observed using fluorescence microscopy. Similar oil droplets were observed by optical (Figure 6A) and fluorescence (Figure 6B) microscopy without and with a filter set, respectively. The CD nanogels (green) are concentrated at the surface of the oil droplet, confirming that the adsorption of the CD nanogels at the oil–water interface stabilizes the emulsion.

### Organization of CD nanogels at the interface

**Figure 7:**
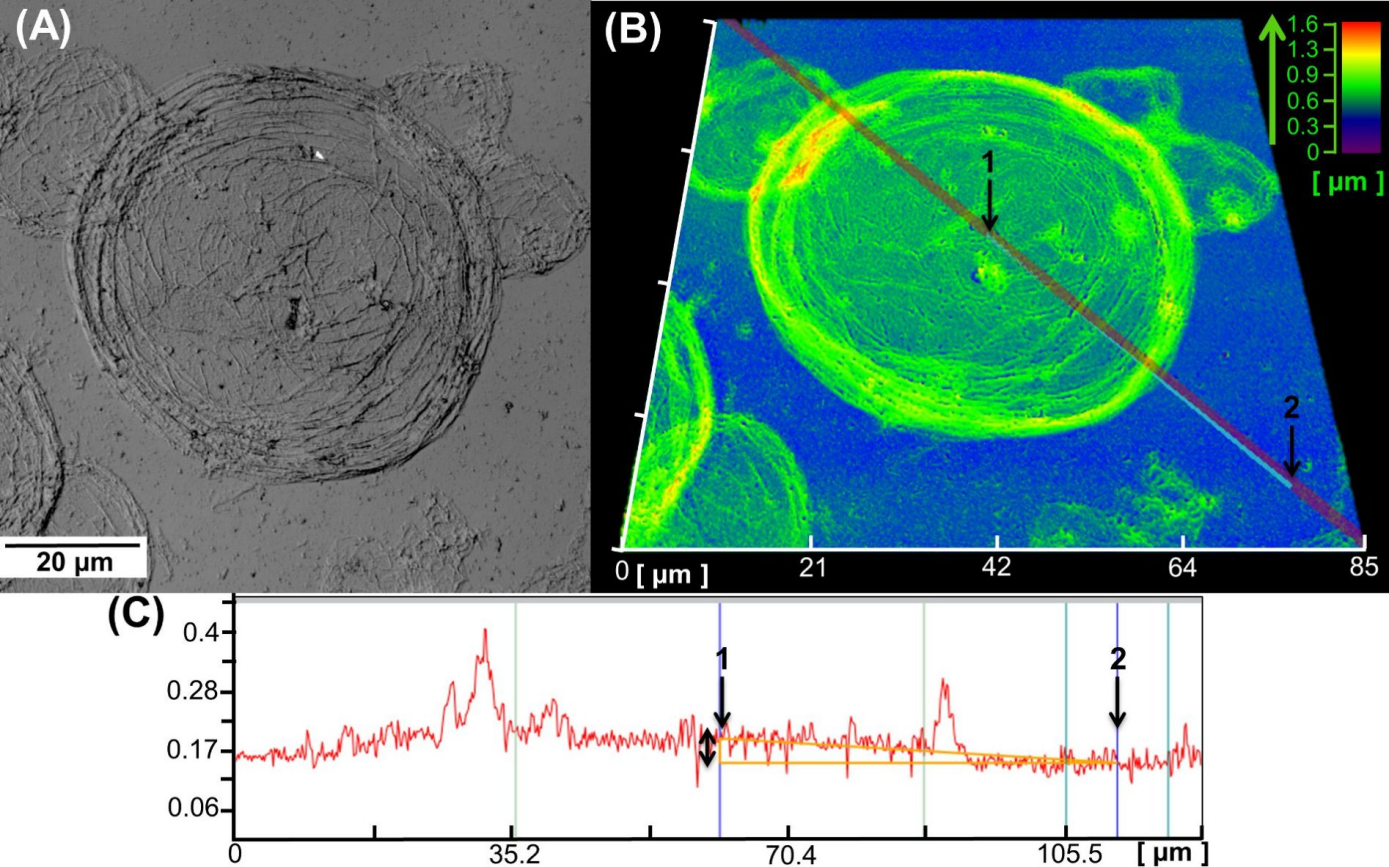
Laser microscope images of the toluene-in-water droplets stabilized by the DM-β-CD/PDI nanogel after evaporation of the toluene cores. (A) Optical image, (B) profile image, and (C) the cross-sectional histogram. Inset arrows and numbers correspond to the orientation for both images (B and C).

To characterize the interfacial structure and to examine the stability of interfacial assembly of the CD nanogels, laser or scanning microscopy observation was performed after evaporation of the oil droplets. Figure 7A,B show the optical and profile images of the toluene-in-water droplets stabilized by DM-β-CD/PDI nanogel after evaporation of the toluene core. The assembled layer of CD nanogel particles maintains the circular shape of the oil droplet, although the three-dimensional spherical structure collapses and flattens during the drying process. The image appears to have a “deflated balloon” structure, indicating that CD nanogel particles can be fused together via interconnections after adsorption at the oil–water interface.

The “deflated balloon” structures, which consist of a CD nanogel assembly, were also observed after the removal of toluene (Figure S5, Supporting Information File 1). The layer thickness of the CD nanogel assembly was assessed by a cross-sectional histogram using a laser microscope (Figure 7C). The estimated layer thickness between the top layer (Figure 7C-1) and the bottom substrate (Figure 7C-2) is about 46 nm, suggesting that the original nanogel particles (30 to 50 nm in diameter) self-assemble at the oil–water interface to form interconnected monolayers. Hence, the Pickering emulsion should be fabricated from the monolayer shell (whose thickness corresponds to the particle diameter) of self-assembled CD nanogels and not a multilayer structure.

**Figure 8:**
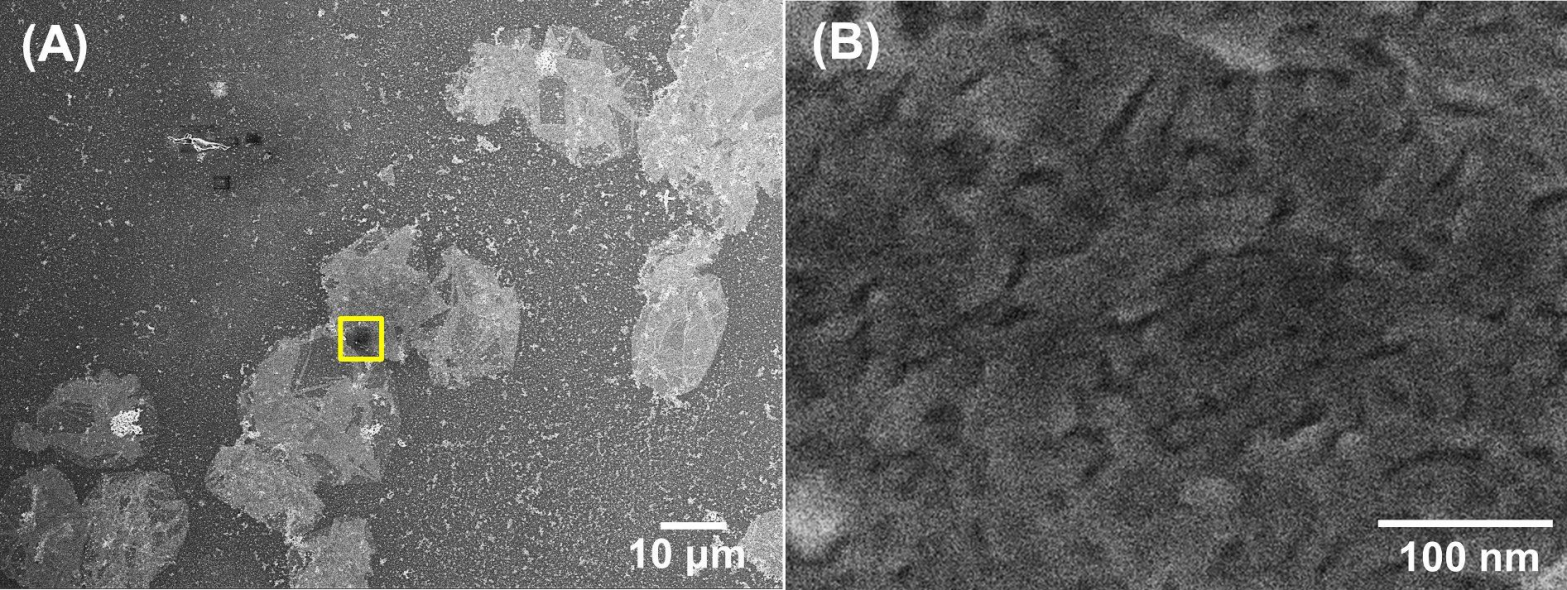
SEM images of the toluene-in-water droplets stabilized by the DM-β-CD/PDI nanogel after evaporation of the toluene core. (B) Magnified image of the square box on (A).

The outermost layer of the CD nanogel assembly was observed using SEM. A number of “deflated balloon” structures appear under low magnification (Figure 8A), while a flattened layer is observed in the magnified image due to the fusion of CD nanogel particles (Figure 8B). The spherical CD nanogel particles are easily inter-penetrable [21] and may form a dense interconnected network (Figure 8B). The strong connectivity results in an interfacial layer, which effectively protects the oil droplets from coalescence.

## Conclusion

Amphiphilic CD nanogels, which are a new class of soft hydrogel nanoparticles, were prepared by crosslinking DM-β-CDs with PDI followed by the immersion in water. The DLS study shows that the primary CD nanogels (30–50 nm in diameter) assemble into larger ones (120 nm in diameter). These CD nanogels show surface-active properties at the air–water interface and function as an effective Pickering emulsion stabilizer at relatively low concentrations (0.05–0.1 wt %). Due to the adsorption property of CD nanogels on droplets, CD nanogels have potential as new building blocks for microcapsules with porous surfaces and stimuli-responsive nanocarriers in storage and/or release systems.

## Experimental

### Materials

Heptakis(2,6-di-*O*-methyl)-β-cyclodextrin (DM-β-CD) was purchased from Nacalai Tesque, Inc. (Japan). DM-β-CD was vacuum dried at 80 °C overnight prior to use. Anhydrous *N,N*-dimethylformamide (DMF) and 1,4-phenylene diisocyanate (PDI) were purchased from Wako Pure Chemical Industries, Ltd. (Japan). Toluene and *n*-dodecane were purchased from Kanto Chemical Co., Ltd. (Japan). Fluorescein-4-isothiocyanate was purchased from Dojin Chemical Co. Ltd. (Japan). The dialysis membrane with a cellulose tube (molecular weight cut-off of 10 kDa) was purchased from the Japan Medical Science Co. Ltd. (Japan) and stored in a moist environment prior to use. Distilled water was used for the purification process. Ultra pure water (Milli-Q) was used for the redispersed solution and emulsion preparation.

### Preparation of CD nanogels

A crosslinked CD polymer was synthesized by reacting DM-β-CD with 1,4-phenylene diisocyanate (PDI) as a crosslinker at a molar feed ratio of diisocyanate to DM-β-CD = 3.0. Dried DM-β-CD (1 g, 0.752 mmol) was reacted with PDI in anhydrous DMF (10 mL) for 12 h at 70 °C under an argon atmosphere. The reaction mixture was dropped into water under ice bath cooling to terminate the reaction. The resulting polymer solution was purified by dialysis for three days using a dialysis membrane to remove unreacted DM-β-CD and PDI. The dialysis water was changed twice daily. Then the aqueous dispersion was lyophilized for three days to yield a white powder as a urethane-crosslinked DM-β-CD polymer. The CD nanogel was prepared by the redispersion of the powder in distilled water with the aid of ultrasonication and stored in a refrigerator until use.

### Characterization of CD nanogels

#### ^1^H NMR spectroscopy

^1^H NMR spectra were recorded in (CD_3_)_2_SO using a 600 MHz JEOL RESONANCE; JNM-ECA600, Delta V5 (Japan) and averaged 16 scans at 25 °C. The chemical shifts of protons associated with different carbon atoms were presented in Table S1, Supporting Information File 1.

#### Dynamic light scattering (DLS)

The particle size distribution was measured at 25 °C using DLS6000HL (Otsuka Electronic Co.) equipped with a He-Ne laser (wavelength 632.8 nm). The mean particle diameter was calculated from the diffusion coefficients using the Stokes–Einstein equation. The particle size distribution was estimated from the scattering intensity function using the histogram analysis method, which was performed with the software supplied by the manufacturer. The weight-average distribution corresponding to each particle size was corrected from the scattering intensity, and the number-average distribution was calculated from the values where the weight-average distribution values were divided by the cube of the particle diameter. All measurements were repeated thrice using an appropriate concentration of the CD nanogel aqueous solution. Milli-Q water was used to dilute the solutions, which were ultrafiltered through a 0.8 μm membrane to remove dust.

#### Electron microscopy

Scanning electron microscopy (SEM) studies were performed using a field emission gun scanning electron microscope (JSM-6700F, JEOL Ltd., Japan) with a beam current of 10 μA at a typical operating voltage of 15 kV. Each Pickering emulsion sample was dried directly onto carbon tape and allowed to dry overnight before being sputter-coated with a thin layer of tungsten. Transmittance electron microscopy (TEM) studies were performed using a JEOL JEM-2100 operating at a voltage of 50 kV in order to clarify the particle morphology. Dilute solutions were prepared and deposited onto a copper grid covered by a carbon membrane at ambient temperature. The electronic contrast of the specimen was enhanced by labeling with ammonium molybdate.

#### Surface tension

The surface tension of the aqueous solution was measured using a CAX-150 (Kyowa Interface Science Co., Ltd) operating in the pendant drop method. The solution was added to a glass syringe after standing for 1 h at 25 °C. The droplet was prepared just before it fell to the ground. The surface (interfacial) tension (γ) was calculated by ds/de method proposed by Andreas [22], which is expressed as γ = *g*(D_e_)^2^Δρ/H where *g* is the gravitational constant, Δρ is the difference between the densities, D_e_ is the equatorial diameter of the drop, H is a correction factor related to the shape factor of the pendant drop (S). S is defined as S = D_s_/D_e_ where D_s_ is the drop diameter measured horizontally at a distance D_e_ away from the apex of the drop. The density between air and water was assumed to be 0.997 g/cm^3^. All measurements were repeated thrice using an appropriate concentration of the CD nanogel in the aqueous solution. The standard deviation (S.D.) was calculated from the average of the five runs.

#### Preparation of a Pickering emulsion

The CD nanogel concentration was adjusted to 0.1 wt % using Milli-Q water. The CD nanogel solution (5.0 mL) was then added to a 13 mL vial, together with the same volume of oil (toluene or *n*-dodecane). The oil/water mixture was homogenized for 1 min using a homogenizer (DIAX 900; Heidolph) at 8000 rpm (25 °C). The resulting emulsion was allowed to stand at 25 °C for 24 h. To confirm the adsorption of the CD nanogels at the oil–water interface in the emulsion, the CD nanogel (0.1 wt %) was labeled with fluorescein-4-isothiocyanate. The aqueous solution of labeled CD nanogel was mixed with *n*-dodecane (Φ_oil_ = 0.1) by hand for 1 min. Then it was allowed to stand for 1 h.

### Characterization of the Pickering emulsion

#### Emulsion stability

The Pickering emulsion stability was evaluated by measuring the volume fraction of the aqueous, emulsion, and oil phases after standing for 24 h. Photographs of the emulsions were taken with a digital camera (FinePix J250, Fujifilm Co., Japan).

#### Drop test

The emulsion type (o/w or w/o) was confirmed by a drop test. An emulsion droplet was placed into water or oil. If the droplet easily spreads and disperses in water, the emulsion’s continuous phase is an aqueous phase. On the other hand, if the droplet remains intact, the continuous phase differs from that of the droplet.

#### Microscope observations

A drop of the diluted emulsion was placed on a glass slide and viewed using an optical microscope (BX50-DIC, OLYMPUS Co., Japan) equipped with a digital camera (D90, Nikon Co., Japan), which was connected to a laptop PC to record the images. The size distribution of the emulsion droplet was determined by measuring the dimensions of 50 droplets from the images recorded.

Fluorescent microscopy was used to observe the emulsion droplet, which was stabilized by the CD nanogel labeled with fluorescein-4-isothiocyanate (FITC). The FITC-labeled CD nanogel was prepared as follow: FITC aqueous solution (1.0 × 10^−5^ M) was added to the CD nanogel aqueous solution (5 mg/mL), and the mixture was stirred at ambient temperature for 24 h. The resulting solution was purified by dialysis for three days using a dialysis membrane (molecular weight cut-off of 10 kDa) to remove free FITC. The aqueous dispersion was lyophilized for three days to yield a powder. Finally, the FITC-labeled CD/PDI polymer was dissolved in water. The fluorescence intensity of the FITC-labeled CD nanogels was detected by fluorescent microscopy (λ_ex_ = 495 nm, λ_em_ = 520 nm). The observations were carried out with filter sets (ex filter 470–490 nm and abs filter 510–550 nm).

The structure of the Pickering emulsion was observed using a laser microscope (OLS4100; OLYMPUS Co., Japan) under atmospheric conditions. The emulsion solution was directly dropped onto the glass slide and dried at ambient temperature. The observation was conducted with a diode laser at 405 nm.

## Supporting Information

File 1 Additional material.
