# Supplementary material for: Preparation of Pickering emulsions through interfacial adsorption by soft cyclodextrin nanogels
Source: Beilstein J Org Chem. 2015 Nov 30;11:2355–64. doi: 10.3762/bjoc.11.257 (PMC4685927; doi:10.3762/bjoc.11.257)
Supplement: File 1 — Additional material. [file Beilstein_J_Org_Chem-11-2355-s001.pdf]

Supporting Information

for

Preparation of Pickering emulsions through interfacial adsorption by soft cyclodextrin nanogels

Shintaro Kawano[1], Toshiyuki Kida*[2], Mitsuru Akashi[2], Hirofumi Sato[1], Motohiro Shizuma[1] and Daisuke Ono*[1]

Address: [1]Biomaterial and Commodity Chemicals Research Division, Osaka Municipal Technical Research Institute, 1-6-50 Morinomiya, Joto-ku, Osaka 536-8553, Japan and [2]Department of Applied Chemistry, Graduate School of Engineering, Osaka University, Suita, Osaka 565-0871, Japan

Email: Toshiyuki Kida - kida@chem.eng.osaka-u.ac.jp; Daisuke Ono - Daiskono@omtri.or.jp,

* Corresponding author

Additional material

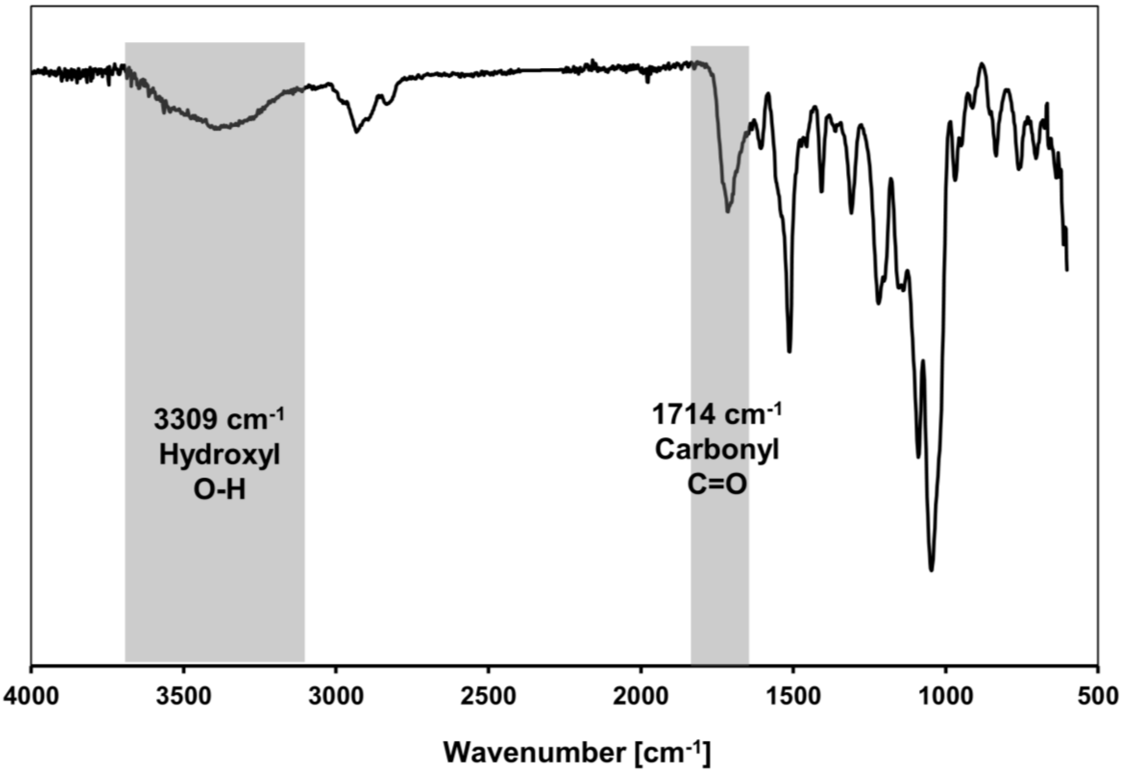

Figure S1: FTIR spectrum of DM-β-CD/PDI polymer.

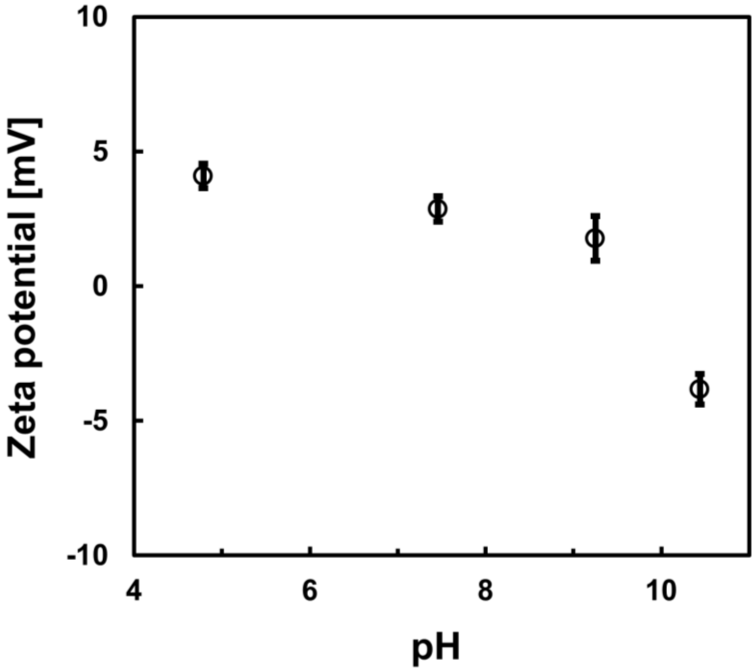

Figure S2: Zeta potential of DM-β-CD/PDI nanogels at various pH levels.

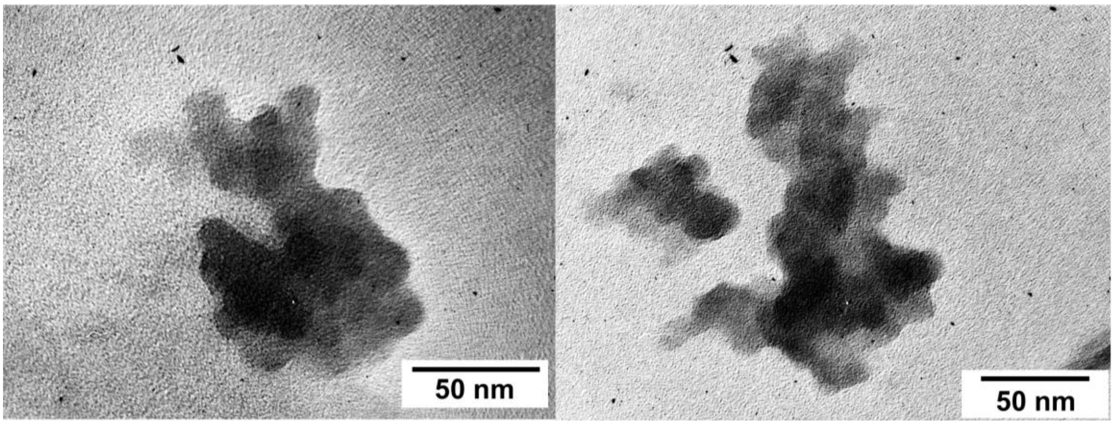

Figure S3: TEM images of self-assembled DM-β-CD/PDI nanogels.

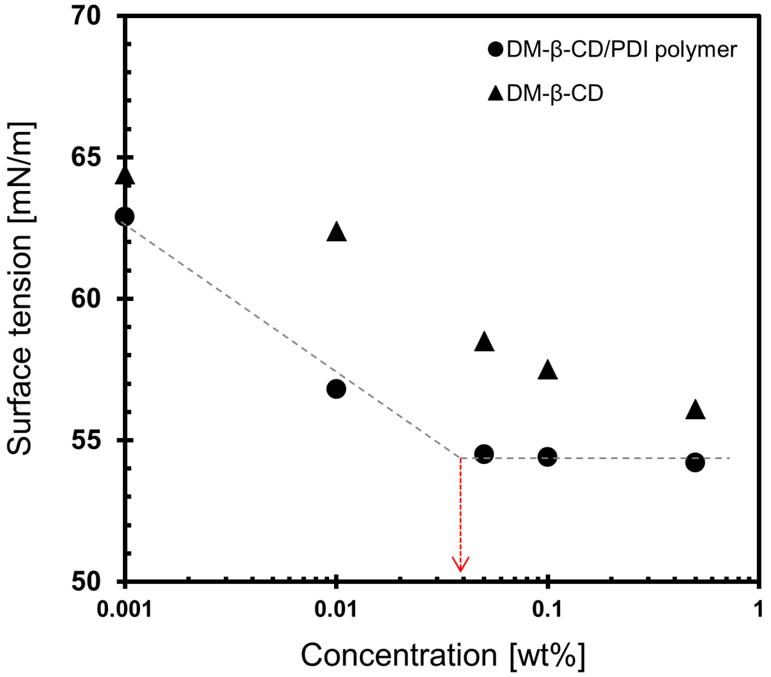

Figure S4: Surface tension vs concentration plots of DM-β-CD/PDI polymer and DM-β-CD aqueous solutions. The arrow shows a critical aggregation concentration of DM-β-CD/PDI polymer solution.

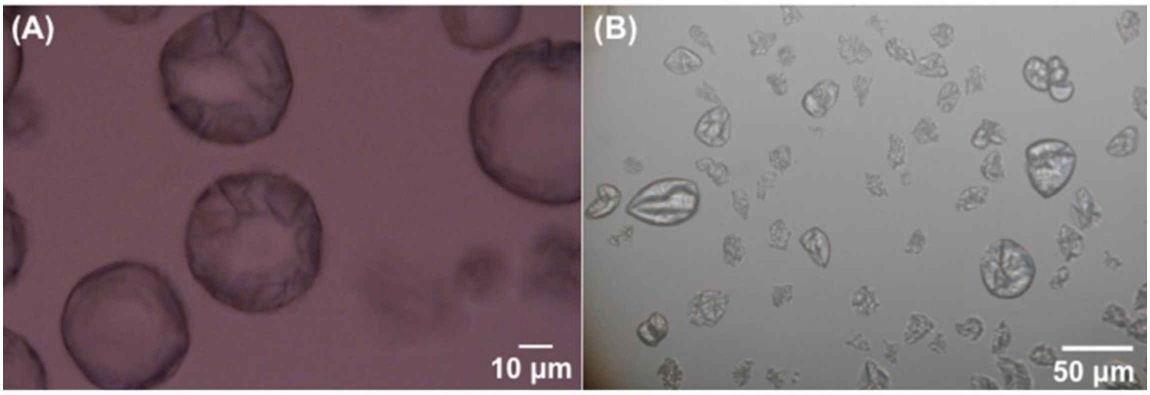

Figure S5: Optical micrograph of the toluene-in-water emulsion stabilized by DM-β-CD/PDI nanogel after the partial removal (A) and evaporation (B) of toluene core.

Table S1: ^{1}H NMR chemical shifts (δ; ppm), integral values and coupling constants (*J*; Hz) of DM-β-CD/PDI polymer and DM-β-CD in $(CD_3)_2SO$.

δ	DM-β-CD/PDI polymer			DM-β-CD		
	Chemical shift (ppm)	Integral	coupling	Chemical shift (ppm)	Integral	coupling
H_1	4.99	1H	br	4.97	1H	m, $J_{1,2}$ = 4.1 Hz
H_2	3.21	1H	br	3.19	1H	dd, $J_{2,3}$ = 9.6 Hz
H_3	3.72	1H	br	3.70	1H	t, $J_{3,4}$ = 9.6 Hz
H_4	3.3[a]	–		3.35[a]	1H	m
H_5	3.54	1H	br	3.68	1H	m
H_6	3.46	2H	br	3.55	2H	m
2-OCH_3	3.3[a]	–		3.50	3H	s
6-OCH_3	3.22	3H	br	3.25	3H	s
phenyl	7.31	4H	br	–	–	–

[a]The signals were overlapped with H_2O.
